# Enhancing GaN/Al_x_Ga_1−x_N-Based Heterojunction Phototransistors: The Role of Graded Base Structures in Performance Improvement

**DOI:** 10.3390/mi15060778

**Published:** 2024-06-13

**Authors:** Lingxia Zhang, Hualong Wu, Chenguang He, Kang Zhang, Yunzhou Liu, Qiao Wang, Longfei He, Wei Zhao, Zhitao Chen

**Affiliations:** 1 School of Physics, Electronics and Intelligent Manufacturing, Huaihua University, Huaihua 418000, China; zhanglingxia0613@163.com; 2 Institute of Semiconductors, Guangdong Academy of Sciences, Guangzhou 510650, China; hechenguang@gdisit.com (C.H.); zhangkang@gdisit.com (K.Z.); liuyunzhou@gdisit.com (Y.L.); wangqiao@gdisit.com (Q.W.); helongfei@gdisit.com (L.H.); zhaowei@gdisit.com (W.Z.); chenzhitao@gdisit.com (Z.C.)

**Keywords:** ultraviolet photodetector, GaN/AlGaN phototransistors, graded base

## Abstract

This research explores the architecture and efficacy of GaN/Al_x_Ga_1−x_N-based heterojunction phototransistors (HPTs) engineered with both a compositionally graded and a doping-graded base. Employing theoretical analysis along with empirical fabrication techniques, HPTs configured with an aluminum compositionally graded base were observed to exhibit a substantial enhancement in current gain. Specifically, theoretical models predicted a 12-fold increase, while experimental evaluations revealed an even more pronounced improvement of approximately 27.9 times compared to conventional GaN base structures. Similarly, HPTs incorporating a doping-graded base demonstrated significant gains, with theoretical predictions indicating a doubling of current gain and experimental assessments showing a 6.1-fold increase. The doping-graded base implements a strategic modulation of hole concentration, ranging from 3.8 × 10^16^ cm^−3^ at the base–emitter interface to 3.8 × 10^17^ cm^−3^ at the base–collector junction. This controlled gradation markedly contributes to the observed enhancements in current gain. The principal mechanism driving these improvements is identified as the increased electron drift within the base, propelled by the intrinsic electric field inherent to both the compositionally and doping-graded structures. These results highlight the potential of such graded base designs in enhancing the performance of GaN/Al_x_Ga_1−x_N HPTs and provide crucial insights for the advancement of future phototransistor technologies.

## 1. Introduction

GaN/AlGaN ultraviolet (UV) photodetectors are highly regarded for their outstanding potential, characterized by their low dark current density and heightened sensitivity. Their capability has spurred extensive research into their applications, notably in missile detection, flame monitoring, and space communications [1,2,3]. Historically, research has predominantly focused on photodetectors without internal gain, such as p-i-n, Schottky, and metal–semiconductor–metal (MSM) photodiodes [4,5,6]. In contrast, although avalanche photodiodes (APDs) offer high quantum efficiency and inherent gain, they require high-voltage biases and are prone to noise due to avalanching [7,8,9,10].

Heterojunction phototransistors (HPTs) present a viable alternative, capable of achieving high current gain with substantially reduced noise compared to APDs, making them suitable candidates for sensitive GaN-based UV detection applications [11]. Achieving high internal gain is critical for advanced UV photodetectors designed to detect low-intensity UV light signals. Consequently, precise engineering of the HPT base’s structural parameters is essential. While previous research has led to advancements in GaN/AlGaN-based HPTs, these studies often overlooked the optimization of the HPT base design [12,13,14,15]. Although L. Sun et al. reported improved performance of an AlGaN HPT with a polarization-doped p-base structure, the underlying mechanism remains unclear, necessitating further systematic research [16].

This paper addresses this research gap by exploring the fabrication and performance analysis of GaN/AlGaN HPTs featuring both a compositionally graded base and a doping-graded base, comparing their efficacy to conventional GaN bases. Both grading approaches are theorized to create a built-in electric field, which facilitates electron transport across the base, thereby reducing base recombination current and significantly boosting common emitter current gain. These modifications are anticipated to substantially enhance the performance of GaN/AlGaN HPTs. Through detailed theoretical analysis and experimental validations, this study aims to elucidate the beneficial impacts of graded base designs, thereby advancing our understanding and application of these devices in high-sensitivity UV detection scenarios.

## 2. Theoretical Calculations

### 2.1. GaN/AlGaN HPTs with a Doping-Graded Base

For GaN/AlGaN HPTs with a doping-graded base, the built-in field resulting from the exponentially graded doping profile is defined as follows:

(1) $$E=\frac{D_{p}}{\mu_{p}}.\frac{1}{p_{B}(x)}.\frac{dp_{B}(x)}{dx}$$

where $D_{p}$ represents the hole diffusion coefficient in the base; $\mu_{p}$ is the hole mobility; and $p_{B}$ is the hole density of the base. Under zero bias, the electron current comprises a drift component and a diffusion component:

(2) $$J_{nE}=-qD_{n}\frac{dn_{B}(x)}{dx}-q\mu_{p}n_{B}(x)E=-\frac{qD_{n}}{N_{B}}\left(n_{B}\frac{dp_{B}}{dx}+N_{B}\frac{dn_{B}}{dx}\right)$$

where the electron diffusion coefficient in the base is denoted by $D_{n}$, and $n_{B}$ represents the electron density of the base. When comparing the electron current in devices with a uniformly doped base, the total current in Equation (2) includes an additional drift component. Multiplying both sides of the equation by $N_{B}\left(x\right)$ and integrating from 0 to *W_B_* yields:

(3) $$\begin{array}{l} J_{nE}\int_{0}^{W_{B}}N_{B}(x)dx=qD_{n}\int_{0}^{W_{B}}\left[n_{B}.\frac{dp_{B}}{dx}+N_{B}\frac{dn_{B}}{dx}\right]dx\\ =qD_{n}n_{iB}^{2}\left[\exp\left(\frac{qV_{BE}}{kT}\right)-1\right] \end{array}$$

(4) $$J_{nE}=\frac{qD_{n}n_{iB}^{2}\left[\exp\left(\frac{qV_{BE}}{kT}\right)-1\right]}{\int_{0}^{W_{B}}N_{B}(x)dx}$$

where $W_{B}$ represents the base width; $n_{iB}$ is the intrinsic carrier density in the base; and $V_{BE}$ denotes the bias voltage of the base–emitter junction. Similarly, the hole current can also be determined as follows:

(5) $$J_{pE}=\frac{qD_{p}n_{iE}^{2}\left[\exp\left(\frac{qV_{BE}}{kT}\right)-1\right]}{\int_{0}^{W_{E}}n_{E}(x)dx}$$

where $W_{E}$ is the emitter width; $n_{iE}$ is the intrinsic carrier density of the base; and $n_{E}$ is the electron density of the emitter. For convenient calculation, the hole concentration distribution is expressed by an exponentially graded doping profile, which we define as follows:

(6) $$\eta =\ln\frac{p_{B}(0)}{p_{B}(W_{B})}$$

where $p_{B}(0)$ and $p_{B}(W_{B})$ are the hole density at *x* = 0 and $x=W_{B}$ in the base, respectively. The base transport factor $\tau_{b}$ can be expressed as follows:

(7) $$\tau_{b}=\frac{Aq\int_{0}^{W_{B}}n_{B}(x)dx}{AJ_{nE}}=\frac{W_{B}^{2}}{2D_{n}}.\frac{2}{\eta }\left[1-\frac{1}{\eta }+\frac{\exp(-\eta )}{\eta }\right]\approx \frac{W_{B}^{2}}{2D_{n}}.\frac{2}{\eta }\left[1-\frac{1}{\eta }\right]$$

where *A* is the cross-sectional area of the emitter–base junction. In the case of a uniform doping base:

(8) $$\tau_{b}=\underset{\eta \to 0}{\lim}\tau_{b}=\frac{W_{B}^{2}}{2D_{n}}$$

Therefore, the amplification factor of the collector current is:

(9) $$\beta^{*}=1-\frac{\tau_{b}}{\tau_{B}}=1-\frac{W_{B}^{2}}{2L_{B}^{2}}.\frac{2}{\eta }\left[1-\frac{1}{\eta }\right]$$

The current and current gain are enhanced compared to a uniform doping base by a factor of:

(10) $$\lambda =\frac{2}{\eta }\left[1-\frac{1}{\eta }\right]$$

Assuming 100% internal quantum efficiency, the emitter injection efficiency and current gain for HPTs with a doping-graded base can be expressed as follows:

(11) $$\gamma =1-\frac{J_{p}}{J_{n}}=1-\frac{D_{pE}}{D_{n}}.\frac{\int_{0}^{W_{b}}N_{B}(x)dx}{W_{E}N_{E}}.\frac{n_{iE}^{2}}{n_{iB}^{2}}=1-\frac{D_{pE}}{D_{n}}.\frac{\int_{0}^{W_{b}}N_{B}(x)dx}{W_{E}N_{E}}.\exp\left(-\frac{\Delta E_{gBE}}{kT}\right)=1-\kappa$$

(12) $$\alpha =\gamma \beta^{*}=1-\left\{\frac{W_{B}^{2}}{2L_{B}^{2}}.\frac{2}{\eta }\left[1-\frac{1}{\eta }\right]+\frac{D_{p}}{D_{n}}.\frac{\int_{0}^{W_{b}}N_{B}(x)dx}{W_{E}N_{E}}.\exp\left(-\frac{\Delta E_{gBE}}{kT}\right)\right\}$$

(13) $$G\approx \beta =\frac{\alpha }{1-\alpha }=\frac{1-\delta }{\delta }\approx \frac{1}{\delta }={\left\{\frac{W_{B}^{2}}{2L_{n}^{2}}.\frac{2}{\eta }\left[1-\frac{1}{\eta }\right]+\frac{D_{p}}{D_{n}}.\frac{\int_{0}^{W_{b}}N_{B}(x)dx}{W_{E}N_{E}}.\exp\left(-\frac{\Delta E_{gBE}}{kT}\right)\right\}}^{-1}$$

where $D_{pE}$ is the hole diffusion coefficient in the emitter, and $\Delta E_{gBE}$ is the bandgap between the emitter and base junction. The factors for a doping-graded base and a traditional GaN base in Equations (11)–(13) can be approximated as follows:

(14) $$\frac{\kappa_{\mathrm{doping}\;\mathrm{graded}}}{\kappa }=\frac{\int_{0}^{W_{b}}N_{B}(x)dx}{N_{B}W_{B}}$$

(15) $$\frac{\sigma_{\mathrm{doping}\;\mathrm{graded}}}{\sigma }=\frac{\left\{\frac{W_{B}^{2}}{2L_{n}^{2}}.\frac{2}{\eta }\left[1-\frac{1}{\eta }\right]+\frac{D_{p}}{D_{n}}.\frac{\int_{0}^{W_{b}}N_{B}(x)dx}{W_{E}N_{E}}.\exp\left(-\frac{\Delta E_{gBE}}{kT}\right)\right\}}{\left\{\frac{W_{B}^{2}}{2L_{n}^{2}}+\frac{D_{p}}{D_{n}}.\frac{N_{B}W_{B}}{W_{E}N_{E}}.\exp\left(-\frac{\Delta E_{gBE}}{kT}\right)\right\}}$$

(16) $$\frac{G_{\mathrm{doping}\;\mathrm{graded}}}{G}\approx \frac{\beta_{\mathrm{doping}\;\mathrm{graded}}}{\beta }={\left(\frac{\sigma_{\mathrm{doping}\;\mathrm{graded}}}{\sigma }\right)}^{-1}$$

For example, the HPT employs a base with exponentially graded magnesium doping, ranging from 3.8 × 10^16^ cm^−3^ at the base–emitter junction to 3.8 × 10^17^ cm^−3^ at the base–collector junction. In this case, η=ln10≈2.3. Therefore, the factor λ in Equation (10) is approximately 0.49, while the factors in Equations (14)–(16) are 0.55, 0.48, and 2, respectively. It can be inferred that the current gain of the HPT can be improved two-fold by introducing a doping-graded base structure.

### 2.2. GaN/AlGaN HPTs with a Compositionally Graded Base

For low-level injection, the majority carriers tend to reach equilibrium:

(17) $$J_{p}=-qD_{P}\frac{dp_{B}(x)}{dx}+q\mu_{p}p_{B}(x)E=0$$

The built-in field caused by a compositionally graded base can be expressed as follows:

(18) $$E\left(x\right)=\frac{D_{P}}{\mu_{p}}.\frac{1}{p_{B}(x)}.\frac{dp_{B}(x)}{dx}=\frac{kT}{q}.\frac{1}{N_{B}(x)}.\frac{dN_{B}(x)}{dx}$$

Thus, the electron current density in the base is expressed as follows:

(19) $$J_{n}=qD_{n}\frac{dn_{B}(x)}{dx}+q\mu_{n}n_{b}(x)E\left(x\right)=qD_{n}\frac{dn_{B}(x)}{dx}+qD_{n}n_{b}(x).\frac{1}{p_{B}(x)}.\frac{dp_{B}(x)}{dx}$$

Both sides of the above equation are multiplied by $p_{B}\left(x\right)$ and integrated from 0 to *W_b_*:

(20) $$J_{n}\int_{0}^{W_{b}}p_{B}(x)dx=qD_{n}\int_{0}^{W_{b}}\left[n_{b}(x).\frac{dp_{B}(x)}{dx}+p_{B}(x)\frac{dn_{B}(x)}{dx}\right]dx=qD_{n}n_{iB}^{2}\left[\exp\left(\frac{qV_{BE}}{kT}\right)-1\right]$$

(21) $$J_{n}=\frac{qD_{n}n_{iB}^{2}\left[\exp\left(\frac{qV_{BE}}{kT}\right)-1\right]}{\int_{0}^{W_{b}}p_{B}(x)dx}$$

where $n_{iB}$ corresponds to the intrinsic concentration in the base, which varies with *x* for a compositionally graded base. The intrinsic concentration at x=0 and $x=W_{B}$ can be described as follows:

(22) $$n_{i0}={\left(N_{C}N_{V}\right)}_{AlGaN}^{\frac{1}{2}}\exp\left(-\frac{E_{gAlGaN}}{2kT}\right)$$

(23) $$n_{iw_{b}}={\left(N_{C}N_{V}\right)}_{GaN}^{\frac{1}{2}}\exp\left(-\frac{E_{gGaN}}{2kT}\right)$$

where $N_{C}$ and $N_{V}$ represent the valence band effective density of states and conduction band effective density of states, respectively. It can be approximated that the alloy composition decreases linearly with *x*. The intrinsic concentration in the base can be approximated as follows:

(24) $$n_{iB}\left(x\right)=n_{iGaN}\sqrt{\frac{{\left(N_{C}N_{V}\right)}_{AlGaN}}{{\left(N_{C}N_{V}\right)}_{GaN}}}\exp\left(-\frac{{\Delta E}_{g}}{2kT}.\frac{x}{W_{b}}\right)$$

where $\Delta E_{g}$ corresponds to the variation in the bandgap of the base; thus:

(25) Jn=qDnexpqVBEkT−1∫0WBniB2(x)WBdx∫0WbpB(x)dx=NCNVAlGaNNCNVGaN.qDnniGaN2expqVBEkT−1pBWBΔEgkT1−exp−ΔEgkT

However, the collector current density $J_{C}$ can be approximated as *J_n_*. Here, we can approximate: $\frac{D_{n(AlGaN)}}{D_{n(GaN)}}\approx 1$, $\frac{{\left(N_{C}N_{V}\right)}_{AlGaN}}{{\left(N_{C}N_{V}\right)}_{GaN}}\approx 1$. Thus, the current and current gain are improved compared to a traditional GaN base by a factor of:

(26) GAl gradedG=JnAlGaNJnGaN=Dn(AlGaN)Dn(GaN).NCNVAlGaNNCNVGaN.ΔEgkT1−exp−ΔEgkT≈ΔEgkT1−exp−ΔEgkT

For instance, in the case of $W_{B}=100\;\mathrm{nm}$, an HPT with a compositionally graded base, transitioning from GaN at the base–emitter junction to Al_0.5_Ga_0.85_N at the base–collector junction, yields a factor in Equation (26) approximating to 12. It can be inferred that the current gain of the HPT can be enhanced by a factor of 12 by introducing a compositionally graded base structure, which is six times higher than that achieved with a doping-graded base.

## 3. Materials and Methods

Based on the theoretical calculations presented above, GaN/AlGaN visible-blind heterojunction phototransistors (HPTs) were fabricated and characterized, featuring a compositionally graded base (Structure A) and a doping-graded base (Structure B). A third structure (Structure C) with a normal base was also fabricated for comparative analysis. The samples utilized in this study were grown on *c*-plane sapphire substrates using a low-pressure metal–organic chemical vapor deposition (MOCVD) system. Precursors included trimethyl-gallium (TMGa), trimethyl-aluminum (TMAl), trimethyl-indium (TMIn), and ammonia (NH_3_), while bis-cyclopentadienyl-magnesium (Cp_2_Mg) and silane (SiH_4_) served as p- and n-type doping sources, respectively. General structural layouts for all samples are provided in Table 1.

For each sample, a 25 nm thick low-temperature GaN nucleation layer was initially deposited, followed by the high-temperature growth of a 1.5 μm thick unintentionally doped (uid) GaN buffer layer. Subsequently, a 1 μm thick n-type GaN layer (with an electron concentration of approximately 3.5 × 10^18^ cm^−3^) was grown as the sub-collector and ohmic-contact layer, followed by a 200 nm thick uid GaN layer (*n* ≈ 8 × 10^16^ cm^−3^) as the collector layer. For Structure A, a 100 nm thick p-type Mg:Al_x_Ga_1−x_N layer (x = 0–0.15, *p* ≈ 3.8 × 10^17^ cm^−3^) was subsequently deposited as the base. The reactor was then cooled to 550 °C in an NH_3_ atmosphere for 30 min to reduce Mg atoms adhering to the chamber wall [13]. A 50 nm thick uid Al_0.15_Ga_0.85_N layer (*n* ≈ 8 × 10^16^ cm^−3^) was grown as the absorbing layer, followed by a 250 nm thick n-type Al_0.25_Ga_0.75_N layer (*n* ≈ 2 × 10^18^ cm^−3^) as the collector contact/window layer. For Structure B, a 100 nm thick p-type Mg:GaN layer was deposited as the base, with the doping concentration gradually increasing and the hole concentration approximating 3.8 × 10^16^–3.8 × 10^17^ cm^−3^. For Structure C, a 100 nm thick p-type Mg:GaN layer (*p* ≈ 3.8 × 10^17^ cm^−3^) was subsequently deposited as the base. The reactor was then cooled to 550 °C in an NH_3_ atmosphere for 30 min to minimize the Mg atoms’ memory effect. A 50 nm thick uid GaN layer (*n* ≈ 8 × 10^16^ cm^−3^) was grown as the absorbing layer, followed by a 250 nm thick n-type Al_0.1_Ga_0.9_N layer (*n* ≈ 2 × 10^18^ cm^−3^) as the collector contact/window layer. Although the device can be scaled to higher voltages by applying a thicker graded structure, this may introduce other issues, such as increased carrier recombination and electrical field non-uniformity, which can lower response speed and optical gain. Details of the layer structures for the three samples are also provided in Table 1.

The uid GaN layer between the emitter and the base serves the purpose of preventing Mg atoms from compensating the emitter layer. This approach aims to enhance Mg redistribution at the base–emitter (B–E) junction, thereby improving the emitter injection efficiency. Simultaneously, the heterojunction formed as the emitter–base junction contributes to a larger built-in potential, facilitating higher injection efficiency and greater gain. It is noteworthy that the heterojunction in all three samples features an energy gap with a 0.1 difference in Al composition. Furthermore, two-dimensional electron gas (2DEG) may be generated in Structures B and C at the GaN/AlGaN interfaces. The lateral 2DEG can impact vertical current transport by enhancing electron mobility, forming high electron concentration channels, reducing interface scattering, and lowering noise levels. The high electron mobility of the 2DEG improves overall device conductivity, while the high-concentration electron channel leads to a more uniform and stable vertical current distribution.

**Figure 1:**
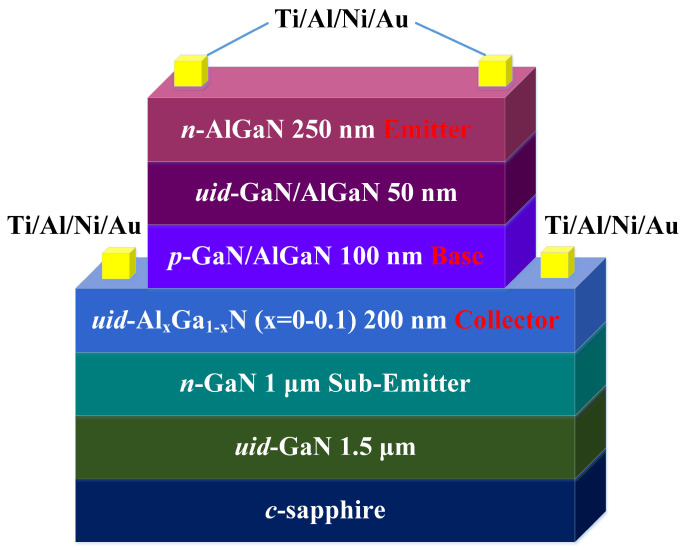
A schematic of the complete GaN/Al_x_Ga_1−x_N HPT structures.

The device fabrication commenced with mesa definition achieved through an inductively coupled plasma (ICP) etching process down to the sub-emitter layer, utilizing BCl_3_/Cl_2_ as gas sources. For the contact electrodes of the emitter and the collector, a Ti/Al/Ni/Au multilayer was formed via electron-beam evaporation. Subsequently, a rapid thermal annealing process was conducted at 830 °C for 30 s in a N_2_ ambient environment, resulting in the formation of ring-shaped ohmic contacts. The fabricated HPTs are two-terminal devices with a floating base, featuring an active area diameter of 150 μm. A schematic diagram illustrating the complete device structure is presented in Figure 1.

The carrier concentrations of the underlayers were determined using Hall measurements conducted on additional samples, where the growth process was interrupted after the underlayers. The structural properties of the as-grown Structures A, B, and C were investigated through high-resolution X-ray diffraction (HRXRD) measurements. Surface morphologies were characterized using a Bruker Dimension Edge atomic force microscope (AFM). Current–voltage (I–V) measurements, both with and without UV illumination, were conducted on the fabricated GaN/Al_0.1_Ga_0.9_N HPTs using a Keithley 4200-SCS semiconductor parameter analyzer. UV illumination was applied from the front side of the device, utilizing an UV lamp with a center wavelength of 357 nm. A UV-enhanced, calibrated Si detector was employed to measure the power density of the incident light. Each I–V curve was recorded after applying a reverse bias of 5 V to eliminate accumulated holes in the base region, which could otherwise lower the quasi-Fermi level and introduce additional gains during testing.

## 4. Results and Discussion

The full-widths at half-maximum (FWHMs) of the HRXRD (002)/(102) plane rocking curves of the GaN epilayers are 246.5/298.2, 230.1/280.4, and 228.2/280.5 arcsecs for Structures A, B, and C, respectively. These values correspond to a total threading dislocation density (TDD) of 4.72 × 10^8^ cm^−2^, 4.18 × 10^8^ cm^−2^, and 4.18 × 10^8^ cm^−2^, respectively [17], indicating a high crystalline quality for all three samples. The crystal quality is comparable to those grown on patterned sapphire substrates (PSSs) [18,19,20]. The TDDs are nearly identical for Structures B and C, while they are slightly larger in Structure A. This difference is attributed to newly generated dislocations caused by lattice mismatches, resulting from the higher Al composition in the base of Structure A.

**Figure 2:**
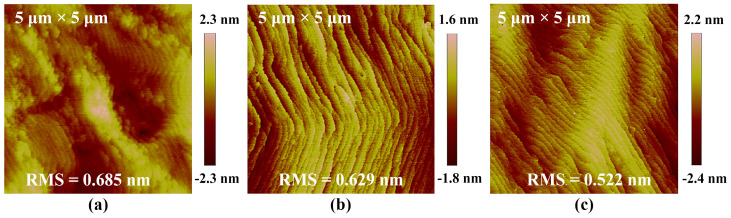
AFM images (5 μm × 5 μm) of Structures (**a**) A, (**b**) B, and (**c**) C.

Figure 2 depicts the surface morphology of the three samples measured by an AFM. Smooth surfaces with distinct atomic steps are evident in all samples. The root mean square (RMS) roughness, defined as the root mean square height difference of peaks and valleys on the surfaces, was measured to be 0.685 nm, 0.629 nm, and 0.522 nm, respectively. These values signify the high crystalline quality of the top AlGaN epilayers. Although the RMS values are similar, the morphological characteristics differ noticeably. Local clusters due to insufficient diffusion of Al adatoms were observed in Figure 2a, indicating degraded crystalline quality due to higher Al mole fractions in Structure A. The mono-atomic steps tend to bunch, and the step edges become blurry in Figure 2b, probably due to flow fluctuations during the growth of the graded layer. Consequently, shifting from a uniform GaN layer to graded structures in the base may slightly degrade the surface morphology. However, whether this trade-off is worthwhile depends on the device’s performance.

**Figure 3:**
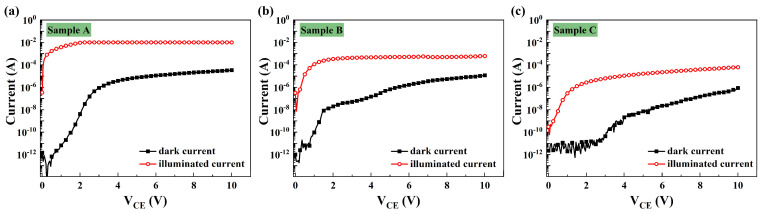
I–V characteristics of (**a**) Structure A, (**b**) Structure B and (**c**) Structure C under dark and UV illuminations.

**Figure 4:**
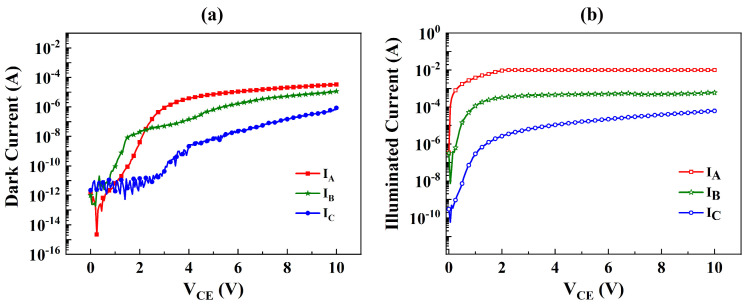
Comparison of (**a**) dark and (**b**) illuminated current of the three structures.

Figure 3 displays the dark current and photocurrent versus the collector–emitter voltage (V_CE_) for the three samples, while Figure 4 provides a comparison of the dark and illuminated current in these samples. In Figure 4a, all samples exhibit typical output characteristics of a bipolar transistor under dark conditions, with an offset voltage ranging from 1.8 to 2.0 V. For V_CE_ values below 2 V, the measured dark collector current (I_Cdark_) remains less than 10 pA. As the V_CE_ increases, the I_Cdark_ exhibits a linear and rapid rise, reaching 0.6 μA, 0.5 μA, and 0.4 μA for Structures A, B, and C, respectively. Subsequently, it approaches saturation more gradually after the V_CE_ exceeds 4.5 V, attributed to the reduction in neutral base width and the subsequent increase in static common emitter current gain with the V_CE_. It is evident that Structure A exhibits a higher dark current, possibly stemming from the compositionally graded base and high TDDs caused by lattice mismatches. Notably, the dark current of Structure C is at least an order of magnitude lower than that of the other two structures.

Figure 4b highlights noticeable differences in illuminated current among the three samples. With an increase in the V_CE_, the illuminated current rises rapidly up to 4.71 × 10^−7^ A, 7.08 × 10^−5^ A, and 5.77 × 10^−4^ A at V_CE_ = 1.2 V for Structures A, B, and C, respectively. Subsequently, it approaches saturation more gradually after the V_CE_ exceeds 2.0 V, attributed to the decrease in neutral base width and the subsequent increase in static common emitter current gain with the V_CE_, which is consistent with the behavior observed in the dark current. Structure A, featuring an Al compositionally graded base, exhibits a more rapid increase and provides the highest illuminated current compared to Structure B with a doping-graded base. Conversely, Structure C, with a conventional base, displays the lowest illuminated current, aligning with the theoretical calculations.

With the assumption of 100% internal quantum efficiency, the current gain is defined as the ratio of charge carriers to the photon flux:

(27) $$G=\frac{hv\Delta I_{c}}{qP_{\mathrm{in}}}$$

where *q* is the electronic charge; Δ*I_C_* (=*I_C_*_ph_ − *I_C_*_dark_) is the net collector photocurrent induced by current injection; *hv* is the incident photon energy; and *P*_in_ is the total incident current power [21,22]. The intensity of the input current power for all samples was consistent. Therefore, the gain varies directly with the Δ*I_C_*:

(28) $$G\propto \Delta I_{c}$$

While V_CE_ = 10 V, for a compositionally graded base, the current gain is improved compared to a GaN base by a factor of:

(29) $$\frac{G_{A}}{G_{C}}=\frac{\Delta I_{cA}}{\Delta I_{cC}}\approx 27.9$$

The experimental current gains of the HPTs with a compositionally graded base are observed to be higher than the theoretical calculations, primarily due to the following factors: In HPTs, incident photons are absorbed, generating electron–hole pairs. These charge carriers are separated by the electric field, drifting towards opposite sides of the junction. Photogenerated holes accumulate in the floating base region, leading to a reduction in the B–E built-in barrier. This barrier lowering facilitates a significant diffusion of electrons across the base to the collector, amplifying the primary photocurrent. For Structure A, under UV illumination with a dominant wavelength of 357 nm, most incident photons traverse the n-Al_0.25_Ga_0.75_N emitter–contact/window layer and p-Al_x_Ga_1−x_N (x = 0–0.15) base layer. They are effectively absorbed in the depletion region of the reverse-biased base–collector junction, generating electron–hole pairs. The photogenerated electrons are efficiently swept to the collector by the strong electric field in the base–collector junction. Conversely, for Structure C, UV light is predominantly absorbed in the GaN layer before reaching the base–collector junction. The photogenerated holes and electrons are separated by the electric field in the emitter–base junction. However, the electric field in the forward-biased emitter–base junction is relatively weak, leading to the possible recombination of photogenerated holes and electrons before separation. This significantly decreases the quantum efficiency. The theoretical bandgap variation (ΔE_g_) of the base can theoretically range from 0 to 0.5 eV. However, an increase in the Al mole fraction of the Al_x_Ga_1−x_N epilayers poses challenges in growing high-crystalline-quality epilayers due to the easier formation of dislocations and uncontrollable doping. Therefore, a tradeoff is necessary between the advantages of an increased built-in electrical field and the disadvantages of increased misfit and threading dislocations. ΔE_g_ should be controlled within a reasonable range, and the recommended value of ΔE_g_ in the HPT with a compositionally graded base is between 0.1 and 0.25 eV.

For a doping-graded base, while V_CE_ = 10 V, the current gain is improved compared to a GaN base by a factor of:

(30) $$\frac{G_{B}}{G_{C}}=\frac{\Delta I_{cB}}{\Delta I_{cC}}\approx 6.1$$

The experimental results for a doping-graded base surpass the theoretical calculations, possibly attributed to a reduced Mg memory effect in Structure B. The phenomenon where Cp_2_Mg tends to be absorbed onto the reactor liners and walls after sources turn off results in the redistribution of Mg atoms in the emitter layer. The comparatively lower Mg concentration in the base of Structure B mitigates the Mg redistribution issue, enhancing emitter injection and consequently increasing the current gain [13]. In a typical bipolar transistor, maintaining a high current gain necessitates the doping concentration in the base to be substantially lower than that in the emitter and higher than that in the collector. Consequently, the factor λ in Equation (10) remains relatively unchanged, primarily limiting the effectiveness of a doping-graded base in enhancing current gain.

## 5. Conclusions

This investigation has focused on GaN/Al_x_Ga_1−x_N-based HPTs featuring both a compositionally graded base and a doping-graded base. Theoretical analyses and empirical fabrications were conducted to evaluate the performance enhancements afforded by these designs. Relative to conventional GaN base structures, HPTs with a compositionally graded base exhibited substantial improvements in current gain, with theoretical predictions and experimental outcomes showing increases by factors of 12 and 27.9, respectively. The observed enhancement correlates with a controlled bandgap variation (ΔE_g_) of 0.15 eV within the graded Al composition of the base.

Additionally, HPTs with a doping-graded base demonstrated significant current gain enhancements, with theoretical and experimental increases by factors of 2 and 6.1, respectively. This improvement is directly linked to the variation in hole concentration, which spans from 3.8 × 10^16^ cm^−3^ at the base–emitter junction to 3.8 × 10^17^ cm^−3^ at the base–collector junction.

Collectively, the results affirm that both doping and compositional gradations within the base structure substantially elevate current gain, with the effects being more pronounced in the compositionally graded bases. These enhancements are principally attributed to the inherent built-in electric fields created by the graded structures. This study not only underscores the efficacy of graded base designs in boosting the performance of GaN/Al_x_Ga_1−x_N HPTs but also contributes valuable insights into optimizing phototransistor designs for enhanced sensitivity and efficiency in UV detection applications.

## Figures and Tables

**Table 1:** Epitaxial parameters of the GaN/AlGaN HPTs on sapphire substrates, where uid refers to being unintentionally dopped.

Sequence	Material	Thickness (μm)	Doping Type	Carrier Concentration (cm^−3^)
7(A)	Al_0.25_Ga_0.75_N	0.25	n	2 × 10^18^
7(B)	Al_0.1_Ga_0.9_N	0.25	n	2 × 10^18^
7(C)	Al_0.1_Ga_0.9_N	0.25	n	2 × 10^18^
6(B,C)	GaN	0.05	uid	8 × 10^16^
6(A)	Al_0.15_Ga_0.85_N	0.05	uid	8 × 10^16^
5(A)	Al_x_Ga_1−x_N (x = 0–0.1 5 )	0.1	p	3.8 × 10^17^
5(B)	GaN	0.1	p	3.8 × 10^16^–3.8 × 10^17^
5(C)	GaN	0.1	p	3.8 × 10^17^
4	GaN	0.2	uid	8 × 10^16^
3	GaN	1	n	3.5 × 10^18^
2	GaN	1.5	uid	8 × 10^16^
1	*c* -plane substrate	-	-	-

## Data Availability

Data are contained within the article.
